# Fatty Acids Intakes and Serum Lipid Profiles: NIPPON DATA90 and the National Nutrition Monitoring

**DOI:** 10.2188/jea.JE20090223

**Published:** 2010-05-05

**Authors:** Yasuyuki Nakamura, Nagako Okuda, Tanvir Chowdhury Turin, Akira Fujiyoshi, Tomonori Okamura, Takehito Hayakawa, Katsushi Yoshita, Katsuyuki Miura, Hirotsugu Ueshima

**Affiliations:** 1 Cardiovascular Epidemiology, Kyoto Women’s University, Kyoto, Japan; 2 Department of Health Science, Shiga University of Medical Science, Ohtsu, Japan; 3 Department of Preventive Cardiology, National Cardiovascular center, Osaka, Japan; 4 Department of Hygiene and Preventive Medicine, Fukushima Medical University, Fukushima, Japan; 5 National Institute of Health and Nutrition, Tokyo, Japan

**Keywords:** food frequency questionnaires, saturated fatty acids, polyunsaturated fatty acids, dietary cholesterol, Keys dietary lipid factor

## Abstract

**Background:**

The National Nutritional Survey in Japan (NNSJ) was initiated in 1946. Using the majority of the participants for NNSJ, the National Survey on Circulatory Disorders (NSCD) has been conducted every 10 year since 1960. We compared fatty acids intakes obtained from NNSJ and serum lipid profiles from NSCD conducted in 1990.

**Methods:**

A total of 8344 community residents (4856 women and 3488 men, age ≥ 30) from 300 randomly selected districts participated in the both surveys in 1990. At baseline, history, physical, and blood biochemical measurement and a nutritional survey were performed. We estimated nutrient intakes of each household member by dividing household intake data proportionally using average intakes by sex and age groups calculated for NNSJ95.

**Results:**

Total fat, saturated fatty acids (SFA), poly-unsaturated fatty acids (PUFA), dietary cholesterol, and Keys dietary lipid factor (KEYS) were inversely associated with age in both men and women (all *P*s < 0.001). In women, age and body mass index (BMI) adjusted serum total cholesterol (TC), high-density lipoprotein cholesterol (HDLc), and low-density lipoprotein cholesterol (LDLc) were positively associated with SFA, total fat intakes (%kcal), and with KEYS (*P* < 0.001). In men, age-BMI adjusted HDLc was not associated with SFA, total fat intakes, and with KEYS factors unlike in women. Other associations were similar to those in women.

**Conclusions:**

The total fatty acids, SFA intakes, and KEYS lipid factor obtained from NNSJ were significantly associated with serum total and LDL cholesterol from the National Survey on Circulatory Disorders conducted in 1990.

The National Nutritional Survey in Japan (NNSJ) was initiated in 1946 under the direction of the supreme commander of the General Headquarters with the main purpose of obtaining factual information on the nutritional health and actual food consumption and food requirements in Japan for emergency food supplies from other countries.^1^ Household-based food consumption data had been collected in order to fulfill the above initial purpose. Recently, the survey has been conducted once every year.

Using the majority of the participants for NNSJ, the National Survey on Circulatory Disorders has been conducted every 10 year in order to obtain cross-sectional data on cardiovascular disease prevalence and risk factors since 1960.^2^ A cohort study based on the National Survey on Circulatory Disorders in 1990^3^ has been names as the National Integrated Project for Prospective Observation of Non-communicable Disease and Its Trends in the Aged (NIPPON DATA90).^4^

In the present study, we compared the fatty acids intakes obtained from NNSJ and serum lipid profiles from the National Survey on Circulatory Disorders conducted in 1990.^3^

## METHODS

### Participants

A total of 8344 community residents (4856 women and 3488 men, aged 30 and greater) from 300 randomly selected districts participated in the National Survey on Circulatory Disorders in 1990. The overall population aged 30 and greater in all districts was 10 956, and the participation rate in this survey was 76.2%. Thus, these participants were thought to be representative of the Japanese population.

### Baseline examination

At baseline, non-fasting blood samples were obtained. The serum was separated and centrifuged soon after blood coagulation. These samples were shipped to one laboratory (SRL, Tokyo) for blood chemistry measurements. Serum triglycerides (TG) and total cholesterol (TC) were measured enzymatically. High-density lipoprotein cholesterol (HDLc) was measured by the precipitation method using heparin-calcium. Lipid measurements were standardized by the Centers for Disease Control/National Heart, Lung, and Blood Institute (CDC-NHLBI) Lipids Standardization Program.^5^

Low-density lipoprotein cholesterol (LDLc) was calculated with the Friedewald formula as follows: LDLc (mg/dL) = TC (mg/dL) − HDLc (mg/dL) − 0.2 × TG (mg/dL).^6^ Body mass index (BMI) was calculated as weight (kg) divided by the square of height (m). Baseline blood pressures were measured by trained observers using a standard mercury sphygmomanometer on the right arm of seated participants.

### Nutritional survey

Food intake survey by weighed food records in three consecutive representative days were conducted by specially trained dietary interviewers. Dietary interviewers visited participants’ houses at least once during the survey. Weekends and holydays were avoided. Up-dated Standard Tables for Food Composition in Japan, 4th edition, with matched fatty acid values and micronutrients, were used to calculate Japanese nutrient intakes.

We estimated nutrient intakes of each household member by dividing household intake data of NNSJ90 conducted in 1990 proportionally using average intakes by sex and age groups calculated for NNSJ95.^7^ The average intakes in NNSJ95 were calculated by a combination method of household-based food weighing record and an approximation of proportions by which family members shared each dish or food in the household. Detailed methods were described.^8^

For each person, means of the estimated individual nutrients from the three days records were used in the analyses. Data are presented as the contribution to total energy intake (%kcal) from total fat, saturated (SFA), polyunsaturated (PUFA), and dietary cholesterol (mg/1000 kcal). Keys dietary lipid score, predictive of serum total cholesterol, was calculated as 1.35 × (2SFA − PUFA) + 1.5 × C^1/2^, where SFA is the percentage of total kilocalories from saturated fatty acids; PUFA, percentage from polyunsaturated fatty acids; C dietary cholesterol in mg/1000 kcal.^9^

### Data analyses

Data were analyzed in men and women separately. BMI, fatty acids intakes, Keys dietary lipid factor and serum lipid profiles were examined by age group. Next, age-BMI adjusted serum lipid profiles by quintile of SFA, Keys dietary lipid factor and total fat intakes using analysis of covariance. To detect differences in continuous variables in groups, analysis of variance was used. The “contrast” option for analysis of variance was used to detect deviation from linearity in the association between continuous variables and the five age groups, and trend *P* was obtained.

## RESULTS

### Fat intakes and serum lipid profiles by age group

Fat intakes, BMI and serum lipid profiles in 4856 women are shown in Table 1. BMI, TC, TG, HDLc and LDLc were significantly positively associated with age up to the 60s age group, then they became slightly lower in the highest age group (all *P*s < 0.001). All the dietary lipid intake variables (total fat, SFA, PUFA, dietary cholesterol intakes and Keys dietary lipid factor) were significantly inversely associated with age (all *P*s < 0.001).

**Table 1.** Fat intake and serum lipid profiles by age group in 4856 women

**Age**	**30–39**		**40–49**		**50–59**		**60–69**		**70–**		**Trend *P***	***P* diff**
***n***	1031		1171		1035		915		704			
**Variable**	**Mean**	**SD**	**Mean**	**SD**	**Mean**	**SD**	**Mean**	**SD**	**Mean**	**SD**		
**BMI (kg/m^2^)**	21.8	*3.0*	22.8	*3.2*	23.4	*3.2*	23.5	*3.6*	22.8	*3.4*	<0.001	<0.001
**Total Fat (%kcal)**	27.3	4.4	25.8	4.2	23.9	4.7	22.4	4.8	21.2	4.7	<0.001	<0.001
**SFA (%kcal)**	7.4	*1.4*	6.9	*1.4*	6.2	*1.4*	5.9	*1.4*	5.6	*1.5*	<0.001	<0.001
**PUFA (%kcal)**	6.5	*1.3*	6.3	*1.3*	6.1	*1.4*	5.7	*1.5*	5.4	*1.4*	<0.001	<0.001
**Chol (mg/1000 kcal)**	209.3	*55.8*	208.2	*55.4*	203.7	*59.5*	187.9	*61.4*	183.7	*60.9*	<0.001	<0.001
**KeysF**	32.8	*5.2*	31.5	*5.1*	29.5	*5.4*	28.5	*5.7*	27.9	*5.8*	<0.001	<0.001
**TC (mg/dl)**	185.7	*32.0*	199.9	*34.7*	218.1	*36.9*	222.4	*38.0*	214.5	*41.9*	<0.001	<0.001
**TG (mg/dl)**	94.8	*55.4*	108.3	*78.3*	134.5	*85.1*	142.7	*86.5*	134.9	*78.6*	<0.001	<0.001
**HDLc (mg/dl)**	59.9	*14.0*	58.9	*14.9*	56.6	*15.4*	54.0	*14.3*	52.6	*15.2*	<0.001	<0.001
**LDLc (mg/dl)**	106.8	*28.3*	119.3	*31.5*	134.5	*32.9*	139.8	*35.0*	134.9	*38.5*	<0.001	<0.001

Values are in mean and *standard deviation*. BMI = body mass index, SFA = dietary saturated fatty acid, PUFA = dietary poly-unsaturated fatty acid, Chol = dietary cholesterol, KeysF = Keys dietary lipid factor, TC = serum total cholesterol concentration, TG = triglycerides concentration, HDLc = high-density lipoprotein cholesterol concentration, LDLc = low-density lipoprotein cholesterol concentration, *P* diff = *P* for difference by analysis of variance.

Fat intakes, BMI and serum lipid profiles in 3422 men are shown in Table 2. BMI, TC, TG, and LDLc had their peaks at age 40s, then they became lower in the higher age groups. All the dietary lipid intake variables were significantly inversely associated with age (all *P*s < 0.001). No difference and trend were observed in HDLc among the age groups.

**Table 2.** Fat intake and serum lipid profiles by age group in 3488 men

**Age**	**30–39**		**40–49**		**50–59**		**60–69**		**70–**		**Trend *P***	***P* diff**
***n***	660		836		793		708		491			
**Variable**	**Mean**	**SD**	**Mean**	**SD**	**Mean**	**SD**	**Mean**	**SD**	**Mean**	**SD**		
**BMI (kg/m^2^)**	22.9	*3.0*	23.4	*2.9*	23.3	*2.8*	22.6	*3.1*	22.0	*3.1*	0.025	<0.001
**Total Fat (%kcal)**	24.6	4.2	23.2	3.9	21.9	4.2	21.1	4.6	20.3	4.5	<0.001	<0.001
**SFA (%kcal)**	6.6	*1.2*	6.3	*1.3*	5.6	*1.3*	5.5	*1.3*	5.5	*1.4*	<0.001	<0.001
**PUFA (%kcal)**	6.0	*1.3*	5.6	*1.1*	5.7	*1.3*	5.4	*1.4*	5.2	*1.3*	<0.001	<0.001
**Chol (mg/1000 kcal)**	190.4	*52.9*	184.8	*48.1*	185.9	*52.9*	180.0	*56.0*	175.7	*58.0*	0.001	<0.001
**KeysF**	30.2	*4.9*	29.6	*4.7*	27.8	*4.9*	27.4	*5.0*	27.4	*5.6*	<0.001	<0.001
**TC (mg/dl)**	196.4	*35.2*	204.5	*36.6*	200.2	*36.6*	197.1	*37.7*	191.4	*36.7*	0.732	<0.001
**TG (mg/dl)**	150.2	*99.0*	164.1	*123.3*	147.4	*106.3*	143.7	*99.2*	123.1	*73.3*	0.049	<0.001
**HDLc (mg/dl)**	50.2	*15.2*	49.7	*14.3*	51.0	*14.7*	50.2	*16.0*	50.1	*15.6*	0.617	0.569
**LDLc (mg/dl)**	116.1	*32.2*	122.0	*33.8*	119.7	*34.8*	118.2	*34.8*	116.6	*33.2*	0.531	0.010

Values are in mean and *standard deviation*. BMI = body mass index, SFA = dietary saturated fatty acid, PUFA = dietary poly-unsaturated fatty acid, Chol = dietary cholesterol, KeysF = Keys dietary lipid factor, TC = serum total cholesterol concentration, TG = triglycerides concentration, HDLc = high-density lipoprotein cholesterol concentration, LDLc = low-density lipoprotein cholesterol concentration. *P* diff = *P* for difference by analysis of variance.

### Age-BMI adjusted serum lipid profiles by quintile of SFA, Keys dietary lipid factor and total fat intakes

Age-BMI adjusted serum lipid profiles by quintile of SFA intake, Keys dietary lipid factor and total fat intake in 4856 women are shown in Table 3. TC, HDLc, and LDLc were significantly positively associated with SFA intake, Keys factor and total fat intake (*P* < 0.001). TG was not associated with SFA intake, Keys factor and total fat intake.

**Table 3.** Age-BMI adjusted serum lipid profiles by quintile of SFA, Keys dietary lipid factor and total fat intakes in 4856 women

**SFA (%kcal)**	**1.35–5.15**		**5.16–6.04**		**6.05–6.82**		**6.83–7.71**		**7.72–14.75**		***P***
***n***	971		970		970		968		975		
	**Mean**	***SE***	**Mean**	***SE***	**Mean**	***SE***	**Mean**	***SE***	**Mean**	***SE***	
**TC (mg/dl)**	198.4	*1.3*	204.8	*1.2*	209.2	*1.2*	209.3	*1.2*	212.8	*1.3*	<0.001
**TG (mg/dl)**	118.6	*2.6*	122.1	*2.5*	123.4	*2.5*	118.9	*2.5*	122.6	*2.6*	0.545
**HDLc (mg/dl)**	54.4	*0.5*	56.0	*0.5*	56.9	*0.5*	57.5	*0.5*	59.4	*0.5*	<0.001
**LDLc (mg/dl)**	120.3	*1.2*	124.4	*1.1*	127.5	*1.1*	128.0	*1.1*	128.9	*1.1*	<0.001

**Keys Factor**	**7.4–25.5**		**25.5–28.8**		**28.9–31.6**		**31.6–34.9**		**34.9–62.5**		
***n***	970		971		971		971		971		
**TC (mg/dl)**	200.7	*1.2*	204.6	*1.2*	207.4	*1.2*	210.0	*1.2*	211.9	*1.2*	<0.001
**TG (mg/dl)**	118.8	*2.6*	121.2	*2.5*	122.8	*2.5*	119.1	*2.5*	123.7	*2.6*	0.579
**HDLc (mg/dl)**	55.6	*0.5*	56.3	*0.5*	56.1	*0.5*	57.9	*0.5*	58.4	*0.5*	<0.001
**LDLc (mg/dl)**	121.3	*1.1*	124.1	*1.1*	126.8	*1.1*	128.3	*1.1*	128.7	*1.1*	<0.001

**Total Fat (%kcal)**	**3.8–20.2**		**20.2–23.1**		**23.1–25.7**		**25.7–28.7**		**28.7–46.1**		
***n***	970		971		971		971		971		
**TC (mg/dl)**	199.2	*1.3*	206.4	*1.2*	208.6	*1.2*	208.8	*1.2*	211.5	*1.2*	<0.001
**TG (mg/dl)**	122.8	*2.7*	124.7	*2.5*	119.8	*2.5*	120.9	*2.5*	117.2	*2.6*	0.326
**HDLc (mg/dl)**	54.0	*0.5*	55.8	*0.5*	57.3	*0.5*	57.7	*0.5*	59.4	*0.5*	<0.001
**LDLc (mg/dl)**	120.6	*1.2*	125.6	*1.1*	127.4	*1.1*	127.0	*1.1*	128.6	*1.1*	<0.001

Values are in mean and *standard error* obtained by age, BMI adjusted analysis of covariance. SFA = dietary saturated fatty acid, KeysF = Keys dietary lipid factor, TC = serum total cholesterol concentration, TG = triglycerides concentration, HDLc = high-density lipoprotein cholesterol concentration, LDLc = low-density lipoprotein cholesterol concentration.

Age-BMI adjusted serum lipid profiles by quintile of SFA, Keys dietary lipid factor and total fat intake in 3488 men are shown in Table 4. TC and LDLc were significantly positively associated with SFA intake, Keys factor and total fat intake (*P* < 0.001). Unlike in women, HDLc was not associated with SFA, total fat intakes, and Keys factor. In men, HDLc was further adjusted for alcohol intake/day. Unlike in women, TG was associated with SFA intake (*P* = 0.032).

**Table 4.** Age-BMI adjusted serum lipid profiles by quintile of SFA, Keys dietary lipid factor and total fat intakes in 3488 men

**SFA (%kcal)**	**1.15–4.73**		**4.74–5.53**		**5.54–6.19**		**6.20–7.00**		**7.01–13.03**		***P***
***n***	971		970		970		968		975		
	**Mean**	***SE***	**Mean**	***SE***	**Mean**	***SE***	**Mean**	***SE***	**Mean**	***SE***	
**TC (mg/dl)**	193.1	*1.4*	195.8	*1.4*	199.7	*1.4*	200.6	*1.4*	204.1	*1.4*	<0.001
**TG (mg/dl)**	144.4	*4.0*	146.6	*3.9*	154.4	*3.9*	153.9	*3.9*	139.6	*4.0*	0.032
HDLc* (mg/dl)	49.2	*0.6*	49.7	*0.5*	50.4	*0.5*	50.8	*0.6*	51.0	*0.6*	0.134
**LDLc (mg/dl)**	113.8	*1.3*	117.3	*1.3*	117.4	*1.3*	122.8	*1.3*	122.8	*1.3*	<0.001

**Keys Factor**	**5.8–24.2**		**24.3–27.1**		**27.1–29.7**		**29.7–32.6**		**32.6–52.7**		
***n***	697		698		698		698		697		
**TC (mg/dl)**	192.9	*1.4*	196.6	*1.4*	200.2	*1.4*	199.3	*1.4*	204.3	*1.4*	<0.001
**TG (mg/dl)**	146.4	*3.9*	150.2	*3.9*	149.5	*3.9*	143.1	*3.9*	149.9	*4.0*	0.653
HDLc* (mg/dl)	50.0	*0.6*	49.9	*0.5*	50.7	*0.5*	49.7	*0.5*	51.0	*0.6*	0.403
**LDLc (mg/dl)**	113.4	*1.3*	116.7	*1.3*	119.4	*1.3*	121.0	*1.3*	123.8	*1.3*	<0.001

**Total Fat (%kcal)**	**5.8–18.7**		**18.7–21.2**		**21.2–23.4**		**23.4–26.0**		**26.0–41.3**		
***n***	697		698		698		698		697		
**TC (mg/dl)**	192.7	*1.4*	198.5	*1.4*	197.5	*1.4*	202.5	*1.4*	201.9	*1.4*	<0.001
**TG (mg/dl)**	146.8	*4.0*	156.5	*3.9*	148.2	*3.9*	145.8	*3.9*	141.6	*4.0*	0.104
HDLc* (mg/dl)	49.2	*0.6*	49.7	*0.5*	50.4	*0.5*	50.8	*0.6*	51.0	*0.6*	0.122
**LDLc (mg/dl)**	113.8	*1.3*	117.3	*1.3*	117.4	*1.3*	122.8	*1.3*	122.8	*1.3*	<0.001

Values are in mean and *standard error* obtained by age, BMI adjusted analysis of covariance. For HDLc*, values are further adjusted by alcohol intake/day. SFA = dietary saturated fatty acid, KeysF = Keys dietary lipid factor, TC = serum total cholesterol concentration, TG = triglycerides concentration, HDLc = high-density lipoprotein cholesterol concentration, LDLc = low-density lipoprotein cholesterol concentration.

## DISCUSSION

One of the most influential ecological studies on diet and coronary heart disease (CHD) was the seven country study by Keys et al that related the mean intake of dietary factors of 16 defined populations in 7 countries to the incidence of CHD in those groups.^10^ The study showed SFA intake as a percentage of calories was strongly correlated with CHD death rates. In another ecological study, a strong association was observed between the percentage of calories from total fat in 12 countries and prevalence of raised atherosclerotic lesions in autopsy cases from the same geographic area.^11^ According to the Third Report of the National Cholesterol Education Program (NCEP) Expert Panel on Detection, Evaluation, and Treatment of High Blood Cholesterol in Adults (Adult Treatment Panel III),^12^ total fat intake was not restricted strictly in order to reduce LDLc, provided SFA are reduced to goal levels (<7%). This guideline is based on that unsaturated fatty acids do not raise LDLc,^13^ and also recommendations that emphasize lower total fat intakes (<30% of total energy) may have led to an overconsumption of carbohydrates, contributing to an increased prevalence of obesity.^14^ Furthermore, very high intakes of carbohydrates (>60% of calories) in overweight/obese persons can aggravates some of the risk factors of the metabolic syndrome.^15^^,^^16^ These latter responses have led some investigators to propose that population with a high prevalence of insulin resistance and the metabolic syndrome should avoid very high carbohydrate diets and should consume relatively more unsaturated fatty acids.^17^ NNSJ90 showed that mean total fat intake was 25.3% of total energy consumed in 1990 in Japan. Mean SFA intake was 6.2% of total energy in our study. Therefore, more than half of Japanese in 1990 were taking SFA not exceeding the upper limit of SFA recommended by the Adult Treatment Panel III.

Age-specific changes in dietary fatty acids and cholesterol observed in the present study are consistent with previous studies.^7^ The sex-specific differences in the associations of HDLc with SFA, total fat intakes and Keys factor observed in the present study appear to be unknown previously. These relations are needed for further analyses.

*In conclusions,* the total fatty acids, SFA intakes, and Key’s dietary lipid factor obtained from NNSJ were significantly associated with serum total and LDL cholesterol from the National Survey on Circulatory Disorders conducted in 1990.
